# Undergraduate Confidence When Undertaking Root Canal Treatment and Their Perception of the Quality of Their Endodontic Education

**DOI:** 10.3390/dj5010001

**Published:** 2016-12-26

**Authors:** James Puryer, Salisha Amin, Maxwell Turner

**Affiliations:** School of Oral and Dental Sciences, Bristol Dental Hospital, Lower Maudlin Street, Bristol BS1 2LY, UK; sa12794@my.bristol.ac.uk (S.A.); mt12282@my.bristol.ac.uk (M.T.)

**Keywords:** undergraduate, endodontics, confidence, teaching

## Abstract

The General Dental Council expects graduating dentists to be competent at treating pulpal disease. Previous studies have found dental undergraduates to have low levels of confidence with respect to endodontic treatments. The aim of this study was to investigate the confidence of undergraduate dental students at the University of Bristol when performing root canal treatment, and to investigate their perception of the quality of their endodontic education. An anonymous questionnaire, based upon one used in a 2015 study at Cardiff University, was distributed to all (*n* = 204) undergraduate students in Years 3–5 at the University of Bristol. The results were analysed using the Statistical Package for the Social Sciences software (SPSS). There was a 59% (*n* = 120) response rate and a significant (*p* < 0.01) difference in confidence levels for root canal treatments (RCTs) completed between these students. All (100%) Year-5 students felt confident in completing anterior RCTs, and 91% felt confident in completing posterior RCTs. The majority (93%) of Year-4 students felt confident in completing anterior RCTs, and 77% felt confident in completing posterior RCTs. Over one-half (56%) of Year-3 students felt confident in anterior RCTs and 17% in posterior RCTs. With respect to the individual stages of RCT (access cavity, cleaning and shaping of root canal system, and obturation/filling), results showed that there was a significant difference (*p* < 0.01) in confidence levels between year groups. Many students thought the amount of time spent on endodontic teaching and the quality of teaching to be satisfactory. Improvements suggested for future endodontic teaching included higher numbers of staff supervision and additional endodontic practice on extracted teeth before seeing patients. There was a strong association between students’ clinical experience and their levels of confidence when completing RCT. Increasing the amount of clinical experience of RCTs could enhance students’ confidence further.

## 1. Introduction

Endodontology is the aspect of dentistry that focuses on the health and the disease status of the dental pulp and peri-radicular tissues [1]. Following a diagnosis of apical periodontitis (the predominant disease of endodontology caused by infection [1]), the aim of treatment is to restore the health of the peri-radicular tissues. The most common way of doing this is by performing root canal treatment (RCT), which aims to either create or maintain (depending on the pre-operative status) an aseptic environment within the root canal system [1]. During their course of study, undergraduate dental students within the UK are expected by the General Dental Council (GDC) to achieve the learning outcomes set out in its document entitled ‘Preparing for Practice’ [2]. Within this document, Learning Outcome 1.14.8 requires dentists, on graduation, to be able to ‘determine the prognosis and undertake appropriate non-surgical treatments to manage pulpal and periradicular disease for uncomplicated deciduous and uncomplicated permanent teeth’ [2]. There are various guidelines available to dental schools that are used to aid the direction of endodontic teaching, including those of the European Society of Endodontology. These state that a dentist upon graduation should be competent at demonstrating ‘a sound theoretical knowledge and understanding of the subject together with an adequate clinical experience to be able to resolve clinical problems encountered independently or without assistance’ [3]. Although the GDC only governs dentistry within the UK, all dental education administrations within the European Union (EU) must reach similar standards to the GDC, and considerable recent research has been undertaken to identify how effectively this has been carried out. Much of this research revealed that undergraduate dental students often fail to perform root canal treatments of adequate quality. A study of Dublin University students’ root canal treatments from 2009 to 2010 showed that inadequate RCTs were performed in 83% of cases [4]. A similar result (87%) was found amongst students at Cardiff University in 2001 [5]. Other analogous studies performed across the EU have produced similar results with the highest percentage of acceptable root fillings reaching only 55% [6,7,8,9]. However, other studies have found contrary results. A 2007 study found that 63% of RCTs performed by undergraduate dental students at the Glasgow dental school were of an acceptable standard [10]. More recently, a study found that over 84% of 258 root canal treatments carried out at Kaunas University in Lithuania were of an adequate standard [11].

The reasons for this variation in standard are unknown and may be due to the ability of the various student cohorts, the clinical techniques used, or the quality of their endodontic teaching. If students feel that their teaching is not of a high standard, their competence and confidence in carrying out RCTs may be affected [12]. However, studies have found that the adequacy of root canal treatment can be highly influenced by various factors including the intra-canal medicament used between visits, the type of tooth, root morphology, pulpal and periapical status, and the radiographic location of voids in the root filling [13,14]. By comparing the primary teaching methods used in different dental schools, it may be possible to determine which teaching methods produce the best quality outcomes. The teaching of endodontics has changed over recent years. Dentists should be providing evidence-based treatments—which allows dentists to keep up to date with the best possible treatments available, as well as incorporating their own clinical skills with patients’ needs, concerns, and values. The type of endodontic teaching available varies between dental schools. Many dental schools take the approach of teaching through problem-based learning, case-based learning, seminars, lectures, tutorials, e-learning, OSCEs, computer-assisted learning, self-teaching, combinations of all these methods (which is common), and more [15]. There remains a strong bias towards the traditional technique of amphitheatre lectures despite there being strong evidence behind there being a need for varied teaching methods in order to achieve the best educational results [12]. A problem-based learning approach has been found to be very beneficial for improving students’ confidence when they go on to perform clinical procedures for themselves [16]. In addition, a case-based discussion method of teaching can increase students’ own perception of how competent they are at carrying out clinical tasks, or, in other words, their confidence in carrying them out [17]. The results of these studies suggest that it would be sensible for dental schools to include either more problem-based learning or case-based discussion teaching within their future courses to compliment or substitute for the more traditional methods that are currently used.

A recent study at Cardiff University explored the quality of endodontic education at its dental school by investigating undergraduate dental students’ perception of how root canal treatment was taught [18]. The study also researched the confidence that dental undergraduates at Cardiff University had when performing either complicated or uncomplicated RCTs clinically. Almost one-half (49%) of students did not feel competent at carrying out root canal treatment on single-rooted teeth, and this figure increased to 74% for multi-rooted teeth. The authors concluded that there was a need for improving the level of endodontic education delivered to the undergraduate dental students at Cardiff University in order to increase their levels of confidence and competence in the subject. Two further studies have found a strong association between the perceived adequacy of undergraduate endodontic education and student confidence in performing RCTs [19,20].

## 2. Aim

- To investigate the confidence of undergraduate dental students at the University of Bristol when carrying out RCT and their perception of the quality of endodontic education.

## 3. Objectives

- To explore the confidence levels of undergraduate dental students when performing root canal treatment;
- To investigate the amount of experience dental students have in the number of root canal treatments undertaken;
- To explore the confidence of dental students when carrying out anterior versus posterior root canal treatment;
- To explore students’ perception of endodontic teaching within Bristol Dental School;
- To investigate how dental students think endodontic education could be improved.

## 4. Methods

Full ethical approval from the Faculty of Medicine and Dentistry Committee for Ethics was obtained prior to the study. Students were informed of the study objectives, that participation was voluntary, that all answers were anonymous, and that consent could be withdrawn at any stage until the questionnaire was returned.

An anonymous cross-sectional survey of all dental undergraduates (*n* = 204) studying in Years 3–5 at the University of Bristol was carried out. There were no exclusion criteria. A questionnaire was developed, based upon the one used in the 2014 Cardiff study, [18] and as the questionnaire had been previously used successfully, no pilot study was undertaken. The survey was undertaken in April 2016. At this time, the Year-3 students would have received a single lecture on endodontics followed by eight sessions in the Clinical Skills Laboratory, and would only be expected to carry out RCTs in single-rooted teeth. Students in Years 4 and 5 would have received a further four endodontic lectures and six further sessions in the Clinical Skills Laboratory, and these students would be expected to carry out RCTs in both single-rooted and multi-rooted teeth. Undergraduate endodontic teaching at Bristol covers both traditional hand-filing techniques and the use of rotary instrumentation using Protaper files (Dentsply Maillefer, Ballaigues, Switzerland).

Data was analysed using Statistical Package for Social Sciences (SPSS). Students’ perception of their confidence at carrying out procedures was determined by use of a series of questions that could be answered with Likert scale values from 1 to 10, with a score of 5 being used as the cut off between ‘confident’ and ‘non-confident’. A Kruskal–Wallis Test determined the difference in students’ confidence between carrying out RCT on anterior and posterior teeth. These tests revealed *p*-values for the data that identified whether or not significant trends between year groups appeared in the data. The tables presented in the Results section include both mode values for the data, which allow comparisons with the recent Cardiff study.

## 5. Results

There was a 59% response rate with *n* = 120 students completing the questionnaire, with response rates from individual years of 62% (Year-3), 65% (Year-4), and 48% (Year-5). 

### 5.1. Endodontic Experience of Completing RCT

Year-3 students had completed a mean of 3.20 RCTs in the Clinical Skills laboratory, with the mean for Years 4 and 5 being 4.42 and 4.38, respectively. In terms of clinical RCTs, the Year-5 students were unsurprisingly more experienced, with a mean of 5.5, compared to means of 0.18 (Year-3) and 1.63 (Year-4), completed RCTs. These results are significant (*p* < 0.01).

### 5.2. Overall Confidence in Completing Root Canal Treatment in a Clinical Setting

There was a significant difference between groups (*p* < 0.01) with respect to confidence levels in completing anterior RCTs (Table 1). All (100%) Year-5 students felt confident (scored > 5) in completing anterior RCTs, with 93% and 56% of Year-4 and Year-3 students, respectively, feeling confident in completing an anterior RCT.

The null hypothesis being ‘the distribution of posterior RCT confidence is the same across categories of year group’ was rejected after carrying out the Kruskal–Wallis Test as *p* < 0.01. In general, students felt less confident in undertaking posterior RCTs compared to anterior RCTs. The majority (91%) of Year-5 students felt confident in performing posterior RCT, whilst 77% of Year-4 and only 17% of Year-3 students felt confident in performing posterior RCT (Table 2).

### 5.3. Perception of Confidence When Performing Different Stages of Root Canal Treatment

Results show that there is a significant (*p* < 0.01) difference between how confident students in different year groups felt at carrying out the three main stages of RCT (access cavity, cleaning and shaping of root canal system, and final obturation). Almost three-quarters (73%) of Year-3 students felt confident at carrying out the access cavity stage with even more (91%) Year-4 and almost all (97%) Year-5 students feeling confident. The results for ‘cleaning and shaping the root canal system’ show that 58% (Year-3), 86% (Year-4), and 97% (Year-5) of students feel confident. Fifty-three per cent (Year-3), 86% (Year-4), and 94% (Year-5) of students feel confident at ‘final obturation’. Table 3 shows that there is an overall significant difference between the years in confidence in performing all stages of RCT (apart from the stages of taking pre-operative, intra-operative, and post-operative radiographs, meaning that there is almost similar confidence in this stage within the year groups). However, between Year-4 and Year-5 students, there was little significant difference in levels of confidence. 

### 5.4. Perception of the Quality of Endodontic Education and How It Can Be Improved

A large proportion (72%) of students rated the amount of time spent on formal endodontic teaching as ‘satisfactory’. In addition, 75% of students thought that the quality of endodontic lectures was ‘satisfactory’, whilst 74% ranked the quality of endodontic practical sessions in the Clinical Skills Laboratory as ‘adequate’.

The students were asked an open question regarding what they thought were positive and negative aspects about their endodontic teaching. Not all of the questionnaires had this question answered and so responses were grouped into themes. Positive themes related to the quality of the teaching staff and the use of transparent practice blocks (endo vu blocks) as being beneficial so that students could receive feedback during practical sessions. Negative themes related to not enough supervision or a lack of supervision leading to a poor structure of the practical sessions in the Clinical Skills Laboratory. Fifteen per cent of students pointed out that the lack of extracted (real) teeth to practice on meant that the Clinical Skills teaching course did not prepare them well enough for carrying out RCTs in a clinical scenario. Some students also pointed out that a lack of patients requiring RCT meant that too long a period had elapsed between their finishing the Clinical Skills teaching course and the first time they treated a patient clinically.

## 6. Discussion

The aim of this study was to investigate the confidence of undergraduate dental students when performing root canal treatment and their perception of the quality of their endodontic education. There was a general improvement in student confidence when performing anterior and posterior RCTs as students progressed through the dental school. This was not surprising as the students gained clinical experience and received further knowledge-based and skills teaching as they progressed from Year 3 to Year 5, as reported in a study conducted at the University of Toronto [21]. This direct relationship between clinical experience and student confidence is demonstrated by Year-3 students (who have on average completed 0.18 RCTs) reporting 37% confidence overall, Year-4 students (average 1.63 completed RCTs) reporting 85% confidence, and Year-5 students (average 4.5 completed RCT’s) reporting 95% overall confidence. It is reassuring that by the time the students started to reach the end of their clinical teaching during Year 5, every student felt confident in performing anterior RCTs, and 91% felt confident in performing posterior RCTs.

The overall confidence in completing the individual stages of RCTs were different between the year groups, again showing that confidence increased with student progression. However, for certain stages of RCT, there appeared to be little difference in confidence between the Year-4 and Year-5 students, suggesting that, by the time students reach Year-4, they have a similar level of confidence to the Year-5 students. This may be attributed to the timing of the study in relating to the undergraduate teaching. The questionnaire was distributed soon after the Year-4 students had completed their second skills course, and so whilst not having the clinical experience of the Year-5 students, the theoretical knowledge would be fresh in their minds. It was unsurprising that the majority of Year-3 students had little confidence in aspects of post and core provision, as this aspect of teaching is not carried out until students are in Year 4.

The results looking at student confidence in relation to the different stages of RCT within the different year groups used mode values so that the results could be easily compared to the Cardiff study [18]. Although the mean does represent the central tendency better than the mode, for comparative reasons, the mode was most appropriate. The Cardiff study found that Year-3 students mostly scored 1 on the confidence scale for the majority of the stages, the majority of Year-4 students scored 4, and Year-5 students scored 7 or 8 [18]. In comparison, Bristol students were indicating confidence scores of 7 (Year-3), 7 (Year-4), and 7 (Year-5) for the majority of stages (Table 3). The reasons for this are unclear, although it does not seem related to the amount of teaching received. Cardiff students appear to have more endodontic teaching (54 h total) compared to Bristol students (43 h total). These differing confidence levels may be related to the differing amounts of direct patient contact between the two schools. Cardiff students had, on average, completed 0, 0.31, and 2.81 RCTs in Years 3–5, respectively, compared to 0.18, 1.63, and 4.5 average completed RCTs for Bristol students in Years 3–5, respectively. Further research could be carried out to explore reasons for these reported confidence levels between the two schools. It is important to remember, however, that confidence (perception of competence) does always directly relate to competence [22]. A student reporting a high level of confidence with a procedure does not necessarily mean that they will be competent at performing it. Overconfident students may put patients at risk by attempting procedures beyond their skill level, whilst although students may have the necessary skills, context and their internal perception of their ability may not give them self-belief (confidence) to carry out the procedure [23]. Both confidence and competence is likely to grow with increased clinical experience, especially if this clinical experience is structured [24]. Although increased clinical experience of RCT does not necessarily mean that a student will become competent, it is essential that undergraduate students receive sufficient clinical exposure to endodontic treatments else they are unlikely to develop either competence or confidence [25]. However, increasing the level of student experience is not always easy with barriers such as a lack of suitable patients and the constraints of an undergraduate teaching curriculum being ever-present [23]. Irrespective of the level of student experience, it is essential for students to develop insight and an accurate self-assessment of their own competence levels and associated confidence such that further training and clinical experience can be sought after graduation where needed. Upon graduation, dentists need the skill to be able to target their ‘weak’ areas through training by using portfolios, reflection, and personal development plans [23].

As a large number of questionnaires were returned without responses in the comments sections, it is difficult to determine if comments made are representative of the population. However, the comments were analysed to look for common themes. Many of the students commented on the good quality of teaching received during the clinical skills sessions, although the high student-to-staff ratio was regularly reported as being an aspect of teaching that could be improved. A higher number of staff devoted to these sessions would allow staff to spend more time with individual students where needed, and would also improve the overall student experience. Many of the responses received related to the equipment used during the practical teaching sessions, and it was found that students thought that the use of transparent ‘endo vu’ practice blocks and ‘Typodont’ teeth were useful learning aides. Most of these comments came from Year-3 students, and we speculate that this is because these resources allow students to receive good feedback on the quality of their work, making them popular during the earlier stages of their endodontic education. However, students from Years 4 and 5 commented that would have liked more opportunities to practice on real extracted teeth. This supports earlier findings as to why students feel endodontic education is of a lower standard than it could perhaps be [26].

Many students at Bristol thought that the amount of formal endodontic teaching received was sufficient, and that teaching was of a good standard, with lectures and practical sessions being of a sufficient standard. This is in contrast to the Cardiff study where endodontic education was found to be ‘lacking’. The reasons for this difference between schools is unknown and, again, would warrant further research. However, in both studies, questionnaire responses highlighted that an increased availability of patients to give increased clinical endodontic experience would be beneficial to student learning. Interestingly, a similar study in 2009 that gathered data on students’ perception of endodontic teaching experience at a university in Brazil drew the same conclusions [27]. A study that audited the minimum amount of RCTs required to be carried out by dental undergraduates prior to qualification throughout 48 dental schools within the European Union (EU) found that students had to complete, on average, RCTs in 17 canals prior to graduation [28]. This quota is far higher than the minimum current requirements of Bristol undergraduates, which is currently five canals, including at least one molar. 

This current study does have some limitations. There may be bias in the results as the views from the 59% of responding students may not be representative of the overall population of dental students across Years 3–5. There was also a higher proportion of responding students from Year 3 compared to Year 5. We speculate that the lower response rate may have been due to the fact that many studies are being undertaken within the School, and students are subjected to ‘survey overload’, which often affects healthcare workers who are usually under great time pressures [29]. In addition, students’ perceptions of confidence levels are subjective, and there was probably individual variation in interpretation as to where on the 1–10 scale the cut off was for being ‘confident’ and ‘not confident’. This most likely accounted for the skewed perception of confidence in posterior RCTs within the Year-3 cohort with, for example, 17% feeling confident in posterior RCTs despite not having been taught posterior endodontic treatment. Furthermore, the questionnaire should have clarified if premolar teeth counted as either ‘anterior’ or ‘posterior’, as this may have led to some inconsistency.

Despite these limitations, we feel that the methodology used and the 59% response rate provide a contemporary benchmark from which further studies could be conducted. Further research could be undertaken to expand the study to investigate confidence levels within undergraduate students at all UK dental schools, and to investigate if different teaching methods, time spent teaching, or the amount of clinical experience has an effect on student confidence.

## 7. Conclusions

Students’ perception of their own confidence at performing RCTs was directly related to their progression as an undergraduate from Years 3 to 5, and it is encouraging that the majority of students approaching graduation felt largely confident in carrying out RCTs in a clinical setting. Student confidence appears directly related to their amount of clinical experience. The standard and quantity of endodontic teaching at Bristol Dental School appears sufficient for its graduates to feel confident at carrying out RCTs, despite some students highlighting areas where the teaching course could be improved. Providing students with increased clinical experience is likely to increase student confidence further, and in addition, it is essential that students develop good self-assessment of their clinical competence and associated confidence levels such that they can seek further post-graduate training where needed.

## Figures and Tables

**Table 1:** The confidence of students in performing anterior root canal treatment (RCT).

How Confident Do You Feel at Performing Uncomplicated Root Canal Treatment on an Anterior Tooth in a Clinical Setting?		Number of Students in Year Group			Total
		Year-3	Year-4	Year-5	
confident in performing anterior RCT	1 not confident at all	5	0	0	5
	2	5	1	0	6
	3	4	0	0	4
	4	6	2	0	8
	5 confident	12	4	2	18
	6	5	13	4	22
	7	5	9	6	20
	8	3	12	10	25
	9	0	2	8	10
	10 extremely confident	0	0	2	2
Total		45	43	32	120

**Table 2:** The confidence of students in performing posterior RCT.

How Confident Do You Feel at Performing Uncomplicated Root Canal Treatment on a Posterior Tooth in a Clinical Setting?		Number of Students in Year Group			Total
		Year 3	Year 4	Year 5	
posterior RCT confidence	1 not confident at all	23	0	0	23
	2	5	4	0	9
	3	3	2	2	7
	4	6	4	1	11
	5 confident	6	6	3	15
	6	2	13	10	25
	7	0	10	8	18
	8	0	3	5	8
	9	0	1	3	4
	10 extremely confident	0	0	0	0
Total		45	43	32	120

**Table 3:** Students’ perception of confidence when performing each stage of uncomplicated RCT.

QUESTION	Mode Values of Confidence			Significance (*p* Value)			
How Confident Do You Feel:	Year 3	Year 4	Year 5	Comparing Year 3 and Year 4	Comparing Year 3 and Year 5	Comparing Year 4 and Year 5	Overall *p* Value
In determining the restorability of a tooth which is endodontically involved?	5	6	8	<0.01	<0.01	0.230	<0.01
At knowing when to refer patients for more complicated endodontic treatment that is beyond your capabilities?	6	6	6	<0.01	<0.01	0.524	<0.01
In understanding and appropriately managing the risks associated with uncomplicated non-surgical root canal treatment?	4	7	6	<0.01	<0.01	0.423	<0.01
At assessing the quality of a root filling post-operatively?	6	7	8	<0.01	<0.01	0.567	<0.01
At providing analgesia to allow you to carry out root canal treatment isolating the tooth?	8	9	10	<0.01	<0.01	0.098	<0.01
At isolating the tooth?	7	9	9	<0.01	<0.01	0.409	<0.01
At preparing the access cavity?	7	8	8	<0.01	<0.01	0.088	<0.01
Determining the working length of a canal?	7	7	8	<0.01	<0.01	0.062	<0.01
Cleaning and shaping the root canal system?	5	7	8	<0.01	<0.01	<0.01	<0.01
Selecting the appropriate irrigant and irrigating the root canal system?	7	8	8	<0.01	<0.01	0.216	<0.01
Placing an inter-appointment dressing?	7	7	10	<0.01	<0.01	<0.01	<0.01
Filling the root canal system?	7	6	8	<0.01	<0.01	0.023	<0.01
Taking pre-operative, intra-operative and post-operative radiographs?	9	7	9	0.835	0.038	0.068	0.035
Interpreting pre-operative, intra-operative and post-operative radiographs?	6	7	8	0.129	<0.01	<0.01	<0.01
Giving post-operative instructions to patients?	6	6	8	<0.01	<0.01	<0.01	<0.01
Determining the correct recall period for a patient?	8	7	9	<0.01	<0.01	<0.01	<0.01
Knowing how to restore a tooth following root canal treatment?	6	7	9	<0.01	<0.01	<0.01	<0.01
Knowing when a post is required to be placed in a root canal to allow tooth restoration?	1	6	9	<0.01	<0.01	<0.01	<0.01
Knowing how to place a post in a root canal and using it to retain a restoration?	1	5	8	<0.01	<0.01	<0.01	<0.01
